# Acceptability of a trial of vaginal progesterone for the prevention of preterm birth among HIV-infected women in Lusaka, Zambia: A mixed methods study

**DOI:** 10.1371/journal.pone.0238748

**Published:** 2020-09-24

**Authors:** Joan T. Price, Chileshe M. Mabula-Bwalya, Bethany L. Freeman, Jessica Carda-Auten, Winifreda M. Phiri, Kasapo Chibwe, Patricia Kantumoya, Bellington Vwalika, Jeffrey S. A. Stringer, Carol E. Golin

**Affiliations:** 1 Division of Global Women’s Health, Department of Obstetrics and Gynecology, University of North Carolina at Chapel Hill, Chapel Hill, North Carolina, United States of America; 2 Department of Obstetrics and Gynaecology, University of Zambia School of Medicine, Lusaka, Zambia; 3 UNC Global Projects – Zambia, Lusaka, Zambia; 4 Department of Health Behavior, University of North Carolina at Chapel Hill, Chapel Hill, North Carolina, United States of America; University of the Witwatersrand, SOUTH AFRICA

## Abstract

Antenatal progesterone prevents preterm birth (PTB) in women with a short cervix or prior PTB in daily vaginal or weekly injectable formulations, respectively. Neither has been tested for the indication of maternal HIV, which is associated with an elevated risk of PTB. The Vaginal Progesterone (VP) Trial was a pilot feasibility study of VP to prevent HIV-related PTB in Lusaka, Zambia. Using mixed methods, we concurrently evaluated the acceptability of the trial and the study product among participants. Over a 1-year period, we enrolled 140 pregnant women living with HIV into a double-masked, placebo-controlled, randomized trial of daily self-administered VP or placebo. We administered an endline questionnaire to all participants and conducted in-depth interviews with 30 participants to assess barriers and facilitators to uptake and retention in the trial and to study product adherence. All interviews were audiotaped, transcribed, translated into English as needed, and independently coded by two analysts to capture emerging themes. Of 131 participants who completed the questionnaire, 128 (98%) reported that nothing was difficult when asked the hardest part about using the study product. When given a hypothetical choice between vaginal and injectable progesterone, 97 (74%) chose vaginal, 31 (24%) injectable, and 3 (2%) stated no preference. Most interviewees reported no difficulties with using the study product; others cited minor side effects and surmountable challenges. Strategies that supported adherence included setting alarms, aligning dosing with antiretrovirals, receiving encouragement from friends and family, sensing a benefit to their unborn baby, and positive feedback from study staff. Participants who reported preference of a vaginal medication over injectable described familiarity with the vaginal product, a fear of needles and resulting pain, and inconvenience of a weekly clinic visit. Those who would prefer weekly injections cited fewer doses to remember. Perceived barriers to study participation included mistrust about the motivations behind research, suspicion of Satanism, and futility or possible harm from a placebo. We report key influences on acceptability of a randomized trial of VP to prevent PTB among HIV-infected women in Zambia, which should inform methods to promote uptake, adherence, and retention in a full-scale trial.

## Introduction

Maternal HIV increases the risk of preterm birth (PTB) [1]. While PTB is a global epidemic, the burden is concentrated in low-resource settings where preventive therapies and life-saving neonatal intensive care are often limited [2]. Inexpensive and scalable interventions are urgently needed to prevent PTB among women at highest risk of delivering prematurely. Antenatal progesterone, available in injectable and vaginal formulations, lowers the risk of PTB in women with some high-risk conditions but has not been evaluated for maternal HIV [3]. We hypothesize that progesterone supplementation could also reduce the PTB risk in women with HIV infection.

Acceptability is a key factor in driving adherence to medical interventions. Previous studies of vaginal microbicides to prevent HIV infection among non-pregnant women in sub-Saharan Africa have suffered from low adherence and substantial discrepancies between self-reported and objective measures of adherence to self-administered vaginal products [4–6]. Barriers to study product adherence in these studies included lack of confidence in study product efficacy, undesirable side effects, interference with sexual behavior, lack of support from partners and family, and stigmatization associated with antiretroviral use [7]. In the setting of pregnancy, women may be more motivated to adhere to self-administered vaginal products to facilitate better outcomes for their babies, as has been shown in some studies of adherence to antiretroviral medications for prevention of perinatal HIV transmission [8]. This motivation may be a result of both an intrinsic desire to have a healthy baby as well as social desirability resulting from high value placed on successful childbearing, regardless of HIV serostatus [9].

Prior to undertaking a full-scale efficacy trial, we performed a pilot feasibility study of vaginal progesterone (VP) for the prevention of PTB among HIV-infected pregnant women in Zambia. Primary quantitative outcomes of the study were study uptake, product adherence, and retention [10]. Alongside the trial, we conducted a mixed methods analysis involving a structured questionnaire among all study participants and in-depth interviews with a subset of participants to (1) identify facilitators and barriers to vaginal study product adherence; (2) understand preferences for a vaginal versus injectable medication, and (3) evaluate the acceptability of a randomized trial with masking and placebo control.

## Methods

### Study design and population

The VP Trial was conducted at the Kamwala District Health Center in Lusaka, Zambia between July 2017 and June 2018. 140 pregnant women were randomized by permuted block design between 16 and 24 weeks of gestation to receive once-daily self-administered vaginal suppository of either progesterone or placebo in a 1:1 ratio.

Before randomization, all participants underwent a baseline questionnaire, physical exam, point-of-care confirmation of HIV seropositivity (Alere Determine HIV-1/2, Abbott Diagnostics), syphilis screening (SD Bioline Syphilis 3.0, Abbott Diagnostics), and hemoglobin testing, plus routine obstetrical ultrasound for gestational age dating and cervical length screening. Following randomization, participants were given a month supply of vaginal study product and a diary in which they were instructed to record each dose taken. Participants received an instructional sheet on correct product use and storage, which was explained verbally by clinic staff at randomization using a sample applicator and suppository, and subseqently as needed. We also provided participants with a discreet carrier, applicators for daily use, and plastic bags to facilitate the return of used applicators. Participants were asked to return to the study clinic every 2 weeks for product resupply and adherence monitoring by review of dose diaries, counting of unused study product, and testing of single-use plastic applicators for vaginal insertion by validated dye stain assay technique (DSA) [11].

At the final antenatal study visit at approximately 36 gestational weeks, participants completed an interviewer-administered questionnaire assessing overall satisfaction with the study, barriers to study product use, preference for vaginal or injectable progesterone, and perceptions of community attitudes towards a medication to prevent PTB. To further explore facilitators and barriers to adherence and trial acceptability, in-depth one-on-one interviews were conducted among 30 trial participants [12, 13]. While adherence, as measured based on the number of used vaginal applicators returned, was high overall in the VP Trial (91% of participants achieved overall adherence ≥80%), purposive sampling was undertaken to enhance representation of lower adherers. To do this, all participants in the VP Trial were classified as having demonstrated overall high adherence, overall low adherence, early low adherence, or late low adherence. The highest or lowest adherers in each group were invited to participate in the semi-structured interview. Interviews were conducted prior to unblinding of study product so randomization group was not a factor in participant selection. For ethical reasons, women were excluded from participation if they had delivered a stillborn baby or if their liveborn neonate had died prior to the time of the interview.

Prior to participation in the VP Trial, women provided written informed consent that included consent for the qualitative interviews. The study protocol, questionnaire, and interview guides were reviewed and approved by the University of North Carolina Institutional Review Board, the University of Zambia Biomedical Research Ethics Committee, the Zambian Medicines Regulatory Authority, and the Zambian National Health Research Authority prior to study initiation.

### Procedures

The questionnaire administered at the final study visit comprised four statements that participants were asked to rate on a Likert scale of five possible options: strongly agree, agree, neutral, disagree, or strongly disagree. Questionnaires included a range of facial illustrations to facilitate comprehension of the answer choices, particularly for illiterate participants. The four statements were: “I am happy I took part in this study,” “I did not mind taking the vaginal medication once a day,” “If I had a choice between a vaginal medication that I give myself every day and an injection in my arm once a week to prevent an early birth, I would prefer to take the vaginal medication,” and “Women in my community would like to take a medication during pregnancy to prevent them from having an early birth.” Questionnaires were translated, and subsequently back-translated to confirm translation accuracy, from English into Nyanja and Bemba and administered in the participant’s preferred language.

Those selected for the qualitative component of the VP Trial were asked to return to the research clinic site to participate in a single 30-minute interview. A trained female staff member conducted each interview in the participant’s preferred language: English, Bemba, or Nyanja. Interview guides posed open-ended questions in the following topical areas: 1) perceived facilitators and barriers to study product use; 2) preferences for daily vaginal versus weekly injectable progesterone; 3) experiences with study visits and staff; 4) perceptions of other participants’ experiences and family and friends’ attitudes towards the study following disclosure of study participation; and (5) perceived community attitudes towards research and, in particular, placebo-controlled trials. Directly following each interview, the interviewer completed a study summary sheet to document the interview length, the overall mood of the interview, and to highlight key observation and findings. Interviews were audiotaped, transcribed, and translated into English as necessary by a research assistant. An independent staff member reviewed each participant’s interview recording, summary, and transcript for quality control; any discrepancies encountered were adjudicated and corrected as necessary.

### Data analysis

Baseline demographic characteristics of the respondents to the quantitative questionnaire and to those who underwent semi-structured interviews were compared to baseline data from all VP Trial participants. We compared categorical and continuous baseline variables using Chi-square and Wilcoxon rank-sum tests of association, respectively. The distribution of responses to the structured questionnaire was compared between randomization groups. Adherence was calculated per participant as the total number of DSA-positive applicators returned to the clinic divided by the number of days between the date of randomization and last antepartum study visit or delivery, whichever was sooner. Participants were then grouped into tertiles of adherence to contextualize qualitative findings. All quantitative analyses were conducted using Stata version 14 (StataCorp, College Station, TX).

The interview transcripts were uploaded to Dedoose (version 8.2.14, SocioCultural Research Consultants LLC, Los Angeles, CA), a qualitative research management tool, to facilitate thematic analysis. A codebook of topical codes was developed based on the research questions, and the codebook was pilot tested with two transcripts and revised, with inductive codes being added, as needed. The final version of the codebook was then applied to the remaining interview transcripts. Transcripts were independently coded by two research team members. Standardized coding guidelines were followed. Coders captured emerging themes and reconciled discrepancies by discussion until they had achieved consensus. Once the coded transcripts were reconciled, code reports were generated from Dedoose for each code and narrative summaries were written. A key summary report was developed that included a narrative description of the themes and sub-themes that emerged and illustrative quotes highlighting each theme. The research team reviewed the key summary report to discuss and confirm the findings, providing an opportunity for the full team to check the consistency and reliability of identified themes.

## Results

Of 140 women randomized in the VP Trial between July 2017 and June 2018, 131 (94%) completed the structured questionnaire (67 active, 64 placebo), 4 (3%) delivered before the scheduled 36-week antenatal visit, and 5 (3%) were lost to follow-up before completing the questionnaire. Baseline characteristics between questionnaire respondents and non-respondents were similar (Table 1).

**Table 1:** Baseline characteristics of participants in VP Trial, questionnaire respondents, and participants interviewed.

Characteristic	All participants N = 140		Questionnaire respondents N = 131		p*	Participants interviewed N = 30		p*
	Median (IQR) or *N* (%)		Median (IQR) or *N* (%)			Median (IQR) or *N* (%)		
Age, years	28	25, 33	28	25, 33	0.222	27	24, 31	0.143
Education, years	8	7,9	8	7,9	0.324	9	6,9	0.799
Either married and/or cohabiting with partner	121	86.4	113	86.3	0.824	27	90.0	0.519
Running water in house	58	41.4	54	41.2	0.849	12	40.0	0.858
Electricity in house	124	88.6	115	87.7	0.265	124	88.6	0.355
Roof material of house					0.949			0.763
Thatch	1	0.7	1	0.8		0	0	
Tin	73	52.1	68	51.9		17	56.7	
Slate or tile	66	47.1	62	47.3		13	43.3	
Cooking fuel used in house					0.521			0.146
Electricity	27	19.3	26	19.9		3	10.0	
Charcoal / Coal	113	80.7	105	80.2		27	90.0	
Flush or pour toilet in house	48	34.3	45	34.4	0.950	9	30.0	0.577
Household assets, (scale 0–16)	8	5,9.5	8	5,10	0.332	7	5,9	0.316
Primigravid	13	9.3	11	8.4	0.167	2	6.7	0.577
BMI, kg/m2	26.2	24.3, 30.1	26.5	24.4, 30.2	0.020	27.3	24.7, 31.2	0.198
Hemoglobin, mg/dL	11.6	10.7, 12.5	11.6	10.8, 12.5	0.112	11.3	10.4, 12.6	0.566
HIV diagnosed prior to pregnancy	97	69.3	91	69.5	0.860	20	66.7	0.726
ART initiated prior to pregnancy	95	67.9	89	67.9	0.937	20	66.7	0.875
Syphilis screen positive	24	17.1	24	18.3	0.158	3	10.0	0.242
UTI (3+ leukocyte esterase or + nitrites on urine dip)	6	4.3	6	4.6	0.512	1	3.3	0.771
Alcohol in pregnancy	17	12.1	17	13.0	0.249	4	13.3	0.822
Tobacco in pregnancy	3	2.0	3	2.3	0.646	0	0.0	0.361
Last sexual activity <24 hours at screening	23	16.4	22	16.8	0.791	5	16.7	0.627
Vaginal washing	99	70.7	92	70.2	0.070	22	73.3	0.381
Last vaginal washing <24 hours at screening, n = 99	93	93.9	86	93.5	0.922	22	100.0	0.610
EGA at screening, weeks	19.9	17.3, 21.9	19.9	17.4, 21.9	0.628	19.5	17.4, 21.9	0.811
Randomized to progesterone	70	50.0	64	48.9	0.301	19	63.3	0.099
Percent adherence (mean ± SD)	94.3	9.4	94.5	8.9	0.797	91.2	11.0	0.022
Lowest tertile	46	33.6	44	33.6		15	50.0	
Middle tertile	46	33.6	45	34.4		7	23.3	
Highest tertile	45	32.9	42	32.1		8	26.7	
EGA at delivery, wks	39	29, 42	39	29, 42	0.041	39	37, 40	0.889
Preterm, <37 weeks	19	14.2	15	11.6		1	3.3	

IQR, interquartile range; ART, antiretroviral therapy; UTI, urinary tract infection; EGA, estimated gestational age; SD, standard deviation.

* *p* values of association compared to the full cohort calculated by chi-square and Wilcoxon rank-sum tests for categorical and continuous variables, respectively.

In-depth interviews were conducted among 30 (21%) participants (19 active, 11 placebo), 15 (33%) women in the lowest tertile of adherence, 7 (15%) in the middle tertile, and 8 (18%) in the highest tertile. Due to purposive oversampling of participants in the lowest tertile of adherence, those who underwent in-depth interviews had lower overall adherence (mean 91.2±11.0) compared to the entire cohort (mean 94.3±9.4). Only 1 (3%) interviewed participant delivered a preterm infant (at 35 gestational weeks), whereas 19 (14%) in the overall cohort did. Although the selection of participants for in-depth interviews occurred prior to unblinding of the treatment arms, those randomized to progesterone were modestly over-represented among interviewees (n = 19; 63%).

### Quantitative questionnaire

**Fig 1:**
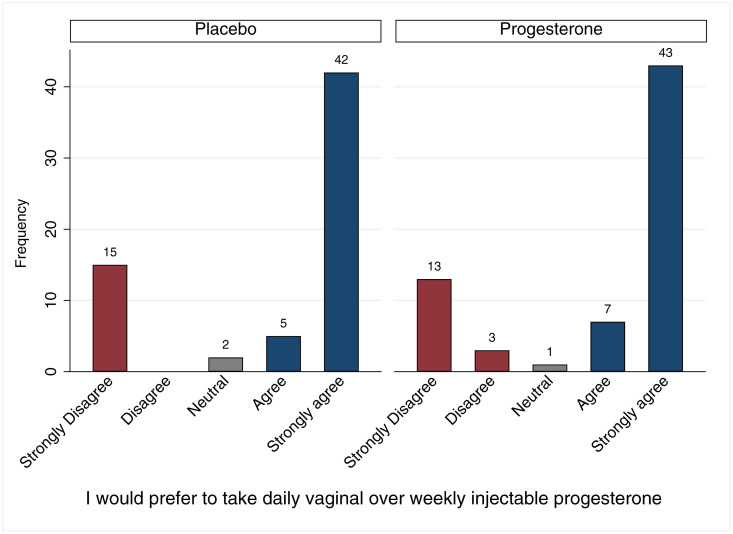
Formulation preferences by randomization group. Reported preferences of daily vaginal versus weekly injectable progesterone formulation were similar by randomized study group among participants completing questionnaire at final antenatal visit in VP Trial, n = 127.

Nearly all participants answered that either they strongly agreed (n = 126; 96%) or agreed (n = 4, 2%) with the statement “I am happy I took part in this study”. Similarly, to the statement “I did not mind taking the vaginal medication once a day”, 118 (90%) responded that they strongly agreed and 12 (9%) agreed. In response to the statement “I would prefer to take the vaginal medication over intramuscular”, 85 (65%) strongly agreed, 12 (9%) agreed, 3 (2%) were neutral, 3 (2%) disagreed, and 28 (21%) strongly disagreed. Distribution of participants across the range of the Likert scale for formulation preference was similar regardless of study randomization group (Fig 1). Finally, to the statement “women in my community would like to take a medication to prevent preterm birth”, 84 (64%) strongly agreed, 31 (24%) agreed, 12 (9%) were neutral, 3 (2%) disagreed, and 1 (1%) strongly disagreed.

### Qualitative interviews

#### Study product use

Participants were asked what it was like to take this medication and if there was anything that made using this medication easy or difficult for them.

*Experiences with study product use*. Most participants interviewed reported experiencing no challenges to study product use. Every participant interviewed said it was easy to understand and follow the verbal and printed instructions on using applicators for inserting the medication. Aspects of product use that participants described as being reasons it was easy for them included the ease of insertion, lack of discomfort when the study product was in place, and the absence of side effects.

1. On my side, I didn’t see any problem inserting the medication, the time and there’s nothing that I had, no bad effects of anything until I delivered. So this program, I was very happy about it.

(29 years old, 100% adherence, progesterone)

Those who did experience side effects reported symptoms such as vaginal itching or abdominal pain, which were typically mild and resolved despite continuation of study product. The frequency of related adverse events was similar between randomization groups [10].

2. …when I started at first, it used to work well. The second one, it started giving me stomach pains. I would feel pain in the stomach, I went to the clinic and they told me that I had sores in the stomach…but I didn’t stop using the medication. I just continued and I felt, everything that I used to feel, the pain even stopped.

(22 years old, 72% adherence, progesterone)

Some participants reported challenges with insertion of the study product, including: getting accustomed to insertion, difficulty inserting the medicine due to increasing belly size, study product melting when exposed to heat, and discomfort caused by bending the applicator during insertion. Notably, all participants reporting these challenges found ways to navigate them, and no participant discontinued use of study product due to challenges or discomfort associated with use.

3. At first, it was very difficult because how to insert the medication, I never had experience, yes, how to put it and everything, until the time I continued using the same medication. I got used [to it] so it became easy for me.

(28 years old, 100% adherence, progesterone)

4. …after the pregnancy became big again, I struggled inserting the medication, it was a problem so I had to get help from my husband.

(23 years old, 85% adherence, progesterone)

Most participants reported experiencing no disruptions to daily life, and there were few reports of disruptions to sexual relations with partners. Some participants described modifying sexual activities while taking the medication generally by timing medication insertion so as not to coincide with intercourse. One participant reported choosing to avoid sex altogether so as not to disturb the product once inserted.

5. …since I started I’ve never had any problem, even when my husband wants to have sex, we would have sex, he wouldn’t even know if I’ve inserted or I haven’t. Even when I go to encourage women about the medicine working, they would say “but in the evening when your husband wants you to have sex, won’t he feel that medication when you insert it?” I tell them he won’t feel it, there’s no problem. That’s what I can say.

(33 years old, 97% adherence, placebo)

6. At first… I was using it at night when sleeping, but (giggles) this man that I live with started being difficult that “why this medication, this, that” so that’s how I changed the time; I started putting at 07:00 hours but still more there wasn’t any problem.

(27 years old, 90% adherence, progesterone)

*Facilitators to study product use*. Interview participants were asked what tactics they developed to help them remember when it was time to insert the vaginal medication daily. The most frequently discussed strategy was to set an alarm, followed by relying on others, staying engaged and interested in their health, synchronizing the vaginal medication with other medications (namely antiretrovirals) or other daily routines, and simply relying on their memory. Some participants cited the internal motivation of wanting to protect their babies as driving their ability to remember to use the product every day.

7. I set an alarm and TV2 news starts at 20:00 hours so I used to know I need to go use the medicine.

(23 years old, 100% adherence, progesterone)

1. What helped me remember about this program, because you know this program, the people in this program, you find that when you meet, we would remind each other about how to put the medication or if you forgot, until birth. I would also see people coming here with babies, such and such. So this thing was not something that I used to forget that’s why I would remember.

(29 years old, 100% adherence, progesterone)

8. Because the time I was taking my medicine [ARVs] is the same time I was inserting the medication. When I take the medication, I would remember that I’m supposed to take the medication and insert it. So I would put the same time.

(26 years old, 97% adherence, placebo)

9. Yes, the other thing which helped me is that, I made this become a part of me because I was told the benefits. So I just followed the benefits and told myself that if I don’t do this, anything can happen, so let me just try and continue until I deliver, so it was part of me.

(27 years old, 100% adherence, progesterone)

*Perceived benefits of study product use*. Participants were asked whether they found anything helpful about the study product. The overwhelming majority reported that the ability to carry their pregnancy to full-term was the greatest perceived benefit of taking the study product, despite not knowing whether they were taking active progesterone or placebo.

10. They taught us that there’s this medicine that is now used to prevent giving birth prematurely. I noticed it benefitted me because I reached nine months and there was no problem.

(25 years old, 100% adherence, placebo)

3. Ok, at first I was told the condition we are in [HIV positive], it’s a 50–50 thing where maybe we can deliver a normal child, like for instance I can reach nine months, sometimes I can have a miscarriage. So it helped me to deliver at a normal time, at nine months. I never experienced anything like bleeding, what or having the same miscarriages talked about, no.

(28 years old, 100% adherence, progesterone)

Others described different ways that they believed the medication benefited their delivery and their health and wellbeing, including safe, healthy deliveries and improvements in feeling sick during pregnancy.

11. I didn’t have any problem even when delivering, there was no need even for a C-section. I delivered well without any problem. Not even stopping at any point.

(21 years old, 56% adherence, progesterone)

12. Ok but it helped me very much because I always have complications when I’m pregnant, I get sick a lot but since I started using this medication, I never fell ill until delivery. I only suffered from low blood pressure.

(30 years old, 95% adherence, progesterone)

Once they had been taking the medicine for a while and, in some cases had completed participation in the study, some participants felt so strongly about the benefits of the medication that they reported talking to others about it and encouraging them to participate in the study.

13. No, I am just encouraging people, those that are doubting that this medication may not work; it can harm the baby. I used this medication and it worked well for me. I’m encouraging all my friends’; they should continue to be coming here to get the medication or the injection.

(24 years old, 92% adherence, progesterone)

#### Preferred formulation

When given the hypothetical option to choose between the daily vaginal regimen of the VP Trial or a weekly injectable form of progesterone [3], the vast majority of participants said they would prefer the vaginal form. Reasons cited for preferring the vaginal route of administration included already being accustomed to the method, fear of injection pain, and the inconvenience of having to visit the clinic weekly for injections. A number of women who indicated that they would not prefer injections stated that they would nonetheless be willing do whatever they could to prevent a premature delivery. The main reason provided by those who stated a preference for an injectable product was to avoid having to remember to insert something every day.

When asked whether other women in their community would choose an injection or vaginal formulation, most participants were reluctant to speculate, saying that responses would differ in accordance with people’s individual preferences.

14. But it depends with a person, what you can manage, so you can choose the injection or the one for inserting. It depends how you know yourself.

(31 years old, 79% adherence, placebo)

#### Experience of study participation

We asked participants about their experience of being involved in the VP Trial and also investigated what they had heard from other participants about their experiences.

*Personal experiences of study participation*. All participants said coming to the clinic was a positive experience and mentioned very few challenges or complications. When asked whether it was easy to return used and unused applicators to the clinic, every participant who responded shared that it was an easy process. Many participants noted motivation to adhere to study procedures and return to the study clinic with their used applicators to receive new medication and because they knew the used applicators would be tested for adherence. Participants also mentioned being supplied with bags to discreetly tote the product home and the used applicators back to the clinic, which made the process easier.

6. Because here [clinic], when you bring back that thing [applicator] here, again they were testing to know if you had used it for the medicine, yes. This is the reason for bringing it back … Like I explained, some women, because of ignorance, they can get the medicine, they may keep it and not use it. Then come back again just like that, get the medicine, not use it and just keep it. So it was ok bringing back that tool because they used to test them so that they know if she used it for the medicine or not. That was what was good.

(27 years old, 90% adherence, progesterone)

10. It wasn’t difficult, it was easy … It was easy because it was easy to carry, everything was nicely packed so it can’t give you any problems. You can’t say you didn’t have anything to use to carry or that you carried it in this way and they fell, no. So it was fine.

(25 years old, 100% adherence, placebo)

When asked what made it easy to return for study appointments, participants named several key factors including: the expectation that they would receive kind, competent care and receive the medication from the clinic; appointment reminders; reimbursement for transportation costs; and only having to travel to the clinic twice a month.

15. When we used to come they cared for us; they cooked for us, gave us some money. They really paid attention to us, cared for us. When I was going to deliver, it was like they were my sisters; they even called when I was … at the labour ward, I was just surprised the phone rang, “where are you?” I responded and they said ok, lets pray for you. I just put the phone down when they said lets pray for you and the baby even came out. So I’m saying they should continue with their hearts for bringing people together because in the past, when someone hears that one is HIV positive, they used to think that’s it, I’m dead. But them-there are those who get depressed, who think too much so when we come here, the way they take care of us, we are happy, there is no problem.

(35 years old, 95% adherence, progesterone)

Another motivation cited by many participants was the ability to check on their health and that of their baby. Many women reported being able to remain motivated or overcome ambivalence about participating after talking with other participants, often those who had been enrolled for a longer period.

When asked what study staff could do to make the study better, nearly every participant felt that their experience with the study and staff was just fine and could not offer any suggestions for improvement. Most participants expressed how grateful they were for the program.

3. Yeah, what I can say is I just thank the same people who brought the same study to help women, they should continue helping them, yeah and just to continue helping people, women who are facing such challenges because I have seen a lot of people going through the same thing. So they should just continue, they shouldn’t stop helping them.

(28 years old, 100% adherence, progesterone)

*Reported experience of other participants*. Study participants recounted a number of different types of conversations when asked what kinds of experiences they heard from other women participating in the study. Most described hearing positive reports from other study participants and, in some cases, described receiving encouragement from other participants to join or continue participation in the study. A number of women reported generally hearing positive experiences about good pregnancy outcomes achieved by the medication.

16. Others when starting like the way we found our friends, others were scared. You would find those who had been there longer encouraging us, “no let’s continue, this program is good, we started a long time. It’s not now when we started.” Even me when I started, I was scared that this program that I have joined, what kind of program is it, yes. But I just saw my friends, encouraging me that no it’s just ok, there isn’t any problem.

(31 years old, 99% adherence, progesterone)

Some participants reported hearing negative experiences from other participants, which were generally about side effects such as fatigue, dizziness, headaches, general malaise, rashes, or vomiting. One participant reported hearing about challenges with discomfort upon insertion of the study medication, another described hearing about other participants who concealed the insertion to prevent their husbands from stopping them from using it, and another heard about ways in which storage could change the medication’s color. Most of these women also heard positive experiences from other participants or countered the negative things they had heard with their own positive experiences of using the medication and so were not swayed from adhering to study product or from continuing in the study.

*Disclosure of study participation to others*. Participants were asked whether or not they told any of their friends or family members about their participation in the study and, if so, how they decided to tell them. They were also asked what they said when they told people about the study, and how these people responded or felt about their participation.

All participants interviewed reported talking to at least one person about their study participation. Roughly one third reported only telling their husband, and one third reported telling their husband and a friend or one family member, most often either a sister or mother. The other third reported telling multiple people including their husband and at least two others, generally a combination of family and friends.

Participants who chose to disclose their involvement in this study to their partners described various reasons for doing so. One described feeling a responsibility to tell her husband because, in the past, he had been honest with her about his HIV status. Two others indicated that they told their husbands because, if they did not, their husbands would wonder where they were going every two weeks, and want to make sure they were safe when traveling. Finally, a couple of participants reported telling their husbands because the study staff encouraged them to do so.

Participants who disclosed their study participation to other family members indicated doing so to ensure they had people to talk to about the study, both to support their decision to participate and in case problems arose. One participant who endorsed telling trusted confidants indicated that she needed to tell these people because it would have been difficult to hide participation in the study, and the other specifically described selecting people to tell based on who she wanted to know about her HIV status.

17. I told my husband, my mother and my friends…I told them because I thought doing things by yourself, you can do something wrong. So it’s better you tell two or three people so that they advise you.

(23 years old, 97% adherence, placebo)

Participants overwhelmingly reported either neutral or positive responses from partners, other family members, and friends. When describing responses from partners, and sometimes even other family members, participants often made it sound like they were seeking and generally received approval. For some, the approval was explicitly linked to a desire to protect the baby and, for others, it was a recognition that when you are HIV positive, special treatment is sometimes necessary.

18. Why I told him because my husband knows my status so it wouldn’t have been nice whereby the baby is born premature, it wouldn’t be nice for me and for him. We both love our baby. So I knew that even if I tell him, he would feel good because it’s for protecting the baby.

(26 years old, 100% adherence, progesterone)

For some participants, the initial response from partners, family, or friends was somewhat hesitant. They expressed concerns or asked questions, and then agreed that this was something worthwhile and safe. Only two participants recounted negative responses from family or friends, who discouraged them to continue participation in the program. Neither of these participants appeared to have been dissuaded from participating by the negative responses.

4. And I told them that I’m participating in a certain program at the clinic where I go. Some, they discouraged me that no they just want to, you know the rumors that go round in our compounds…the medicines they are giving you can infect the child, the child may be born dead or the child may be born with sores or born with different diseases. Sometimes maybe they’re initiating your child into Satanism, just a lot of things … When I was starting, they were not comfortable. Each time I say, no I’m going to the clinic for that study that I talked about, they would always discourage me but as time went by, they started encouraging me and when the child was born, they started appreciating that the program was good.

(23 years old, 85% adherence, progesterone)

#### Community perceptions about participation in research

Participants were asked why some women in their community might want to take part in research studies and what women like about participating in research studies. As was the case when asked about preferred route of study drug administration, many participants had difficulty answering this question on behalf of others. Instead, most responded with the reasons they themselves believed participating in research is important, or with what they had found to be helpful about this study. Among those who did respond to the question, most indicated that women would participate in studies primarily to protect the health of their baby or, more generally, to fix a problem.

4. Yes, some may want to be in research studies just so that they can help the near future because the way you had started on us here, you are not helping us but you are helping our children and our children’s children.

(23 years old, 85% adherence, progesterone)

Others thought that women might participate to learn new things, to receive something free, or because the study was recommended by someone they knew. Some participants described specific aspects of the study that would be appealing to other women, such as the welcoming nature of the clinic, where staff treated patients well and information is kept confidential; and the fact that transportation and food was provided.

19. …the people who are found here, they are not rude or anything, they welcome us in a good way, and they relate with us the way one is supposed to relate with a fellow human being… they don’t shout at anyone.

(33 years old, 97% adherence, placebo)

Participants were further asked why some women in their community might not want to take part in research studies, and what women disliked about participating in research studies. The most commonly cited reasons were fear and suspicion of research, especially research offering a new medication. Suspicion that the research was associated with Satanism was repeatedly cited. Some reported that women may not believe they need this medication to have a full term delivery or that if the study medication were legitimate, it would be offered universally, and not only in one clinic. A lack of information about research, or about the experience of participating in research was also cited, as well as fear based on previous negative experiences of treatment by health center staff.

11. Because we think differently; others say no maybe these people are taking advantage of you. We view things differently…they just say other things like what kind of a clinic gives money to patients, they must be Satanist. They say a lot of things just to discourage you…

(21 years old, 56% adherence, progesterone)

Concerns about HIV status disclosure and of censure or restriction from a partner or others were also given as deterrents to participation in research studies. Participants also speculated that other women may not want to insert the medication vaginally, and reported the belief other women might be fearful of the negative effects the medication may have on them or their baby. It was also suggested that women in the community might not be motivated to undertake daily insertion of the study medication, or make the fortnightly trips to the clinic for study visits, due to feelings that study participation would be a waste of time.

20. Yes, I told a certain friend but she refused. She was also pregnant and she’s also on treatment [ART]. So I tried to tell her about this program, she refused “no, I can’t manage”. But I explained to her how good it is, I explained. So she said I can’t manage, that ok it’s good but I can’t manage.

(32 years old, 69% adherence, placebo)

When asked generally about reasons that others might refuse to participate, not a single participant described any concerns related to the placebo or the “medicine without power.” When asked specifically about concerns surrounding a placebo medication, many participants were able to speculate about potential concerns. Participants suggested that other women in the community would want to be assured that they were receiving something that would be effective. Others speculated on concerns that the placebo would have negative effects for them or the baby. A couple of participants described a belief that while women might agree to participate, they might be fearful or angry if they were given the placebo and had negative outcomes.

21. …like the way they teach that there is medication that is strong and the one that is not strong, others may be scared that I might get medication that is not strong, maybe even have a miscarriage. Others say maybe I can get medication, because that’s like try your luck, like that you get but you can’t know what you have gotten because from that same medication, maybe the medication I got has nothing. So others may have that fear.

(30 years old, 97% adherence, progesterone)

22. If someone gets the medicine and discovers that it has an effect that person would be happy, but if someone gets medicine without an effect, that person would return that medicine and shout at you for giving her something which hasn’t helped her, others would get upset.

(21 years old, 80% adherence, progesterone)

## Discussion

In this mixed methods study among women participating in a randomized feasibility trial of vaginal progesterone to prevent HIV-related preterm birth in Zambia, we found high acceptability of study participation and product use, as well as high levels of product adherence. Key facilitators to study product adherence included receiving encouragement from friends and family, sensing a potential benefit to their unborn baby, and feeling valued and cared about by study staff. Challenges to product adherence were uncommonly reported but included physical barriers to use and disruptions to daily life. Participants reported perceived potential barriers to study participation by other women in the community that included mistrust of the motivations behind the study and research in general, suspicion of Satanism, and futility or possible harm from a placebo. While adherence was overall high in our pilot study and may not be generalizable to a non-study setting, facilitators and barriers identified during this pilot study should inform study staff training, as well as participant and community educational efforts to optimize uptake, adherence, and retention in a full-scale trial.

Previous studies of self-administered vaginal product for the prevention of HIV in non-pregnant African women reported barriers to both adherence and acceptability. Participants described unwanted side effects, interference with sexual behavior, and fear of misattribution of HIV infection if taking antiretroviral medication for prevention [4, 7]. While the latter barrier was not directly applicable to our population of HIV-infected women, we did not find fear of HIV stigmatization or disclosure through study participation to be a strong barrier to product use or participation in general among our participants. Instead, high adherence and acceptability of vaginal product in this study seemed to be driven by a strong motivation to prevent poor birth outcomes that likely counteracted most undesirable side effects, risk of disclosure, and stigmatization. Additionally, the salience of supportive relationships with clinical research staff as motivation for adherence and retention in the VP Trial is consistent with findings from HIV prevention studies in the region, in which women reported attending the study clinic to access quality health services even when not adhering to study product [14, 15]. In future studies, community and participant education efforts should highlight the potential benefit to the pregnancy and unborn baby to encourage study participation, regular attendance at clinic visits, and study product adherence.

While interviewees in this study did not explicitly communicate that a risk of HIV disclosure and stigmatization was a barrier to their own participation, some noted that other women in their community might have faced these barriers when deciding whether to participate. Some participants also reported deciding whom to tell about their participation based on willingness to disclose their HIV status. Although HIV status disclosure to male partners and family members is linked to a number of health indicators including adherence to ART and clinic visits [16–18], these benefits need to be evaluated against potential significant risks for social stigmatization and even intimate partner violence [19]. Since the opinions of family and friends appeared to be very important to study participants’ decisions to enroll and continue in our study, peer and partner involvement will be critical in future research studies and programmatic implementation.

Although we interviewed only women who enrolled in our study and not those who declined, by asking participants to describe barriers other women in their community may face when deciding to participate in research we were able to discover a number of themes that may shed light on barriers future trials may encounter. The most common barrier to study participation noted was mistrust of research in general and explicit suspicion of its relation to Satanism. This finding has been reported in other studies in Zambia, in which community beliefs discourage research participation particularly when it includes phlebotomy due to the fear that researchers use blood samples for Satanic rituals [20, 21]. In addition to blood and biological specimen collection, free non-standard medical services and travel reimbursements are given particular suspicion, as these perceived benefits might be viewed as payment for blood [18]. Additionally, masked placebo-controlled randomized trial designs may cause concerns about taking a study product—whether active or placebo—that is unproven, ineffective, or could even cause harm. Particularly in transnational and transcultural research, the notion of equipoise in placebo randomization must be assured to both the scientific and local communities [22]. While these fears may be individually surmountable to those who agree to participate, study teams in Zambia need to vigilantly identify and address negative perceptions of research programs in the local community prior to implementation and throughout the duration of the study.

In the current study, adherence was measured both by dye stain assay of returned applicators and by participant report via dose diaries. In the primary analysis, we found that participant-completed dose diaries had poor specificity in correctly noting non-adherence when applicators tested negative by DSA [10]. Many interviewed participants revealed using a range of self-reminder strategies such as setting alarms or synchronizing the vaginal study product with other medications or other daily routines, while the dose diaries were not reported to remind or support adherence at all. We noted instead that many participants were motivated by the knowledge that their used applicators would be tested for correct study product use. Previous studies of ART adherence in sub-Saharan Africa have determined that dose diaries for monitoring study product use are likely more accurate than participant recall, but their utility as a direct facilitator of adherence is unknown [23]. Additionally, lower literacy skills among some participants can pose unintended barriers to diary-based documentation of study product use and to product adherence itself [24]. We conclude that a full-scale trial would benefit instead from implementing DSA of returned applicators both to encourage and to objectively monitor study product adherence.

Use of the vaginal study product posed barriers to participants in its physical use, timing of insertion around sexual intercourse, and problems with the product melting. Based on our findings of participants’ reported experiences in the pilot study despite written and verbal instructions, future verbal instructions and written materials should give explicit tips to overcome difficulties with inserting the product particularly in advanced gestation, and for timing product insertion around sexual intercourse and routine vaginal washing practices. Individualized, motivational interviewing, which has been used in other studies of vaginal product to support participants in overcoming obstacles to product use [25], could also be adopted in future trials of progesterone in pregnancy. Finally, although participants were given insulated carrying bags for product storage and instructed to return to the study clinic to exchange any damaged study product, pharmacy staff confirmed that a number of participants returned melted product in October, the hottest month in Zambia. In a large-scale trial, interventions to prevent the study product from melting during the summer could include dispensing reusable frozen gel packs with study product, compounding the suppository using a base with a slightly higher melting point, or using a gel formulation instead.

While multiple formulations of antenatal progesterone supplementation exist, when presented with the choice of daily self-administered vaginal suppositories over weekly injections, most participants in the VP Trial reported a preference of vaginal progesterone. This finding contrasts with a previous study performed in Zambia among 147 HIV-infected and uninfected pregnant women that demonstrated preference of an intramuscular formulation (61%) over either vaginal gel (25%) or vaginal tablet (15%) [26]. However, this difference may be attributed to a familiarity bias since that study was performed among women who had no experience with any of the progesterone formulations, whereas our participants underwent qualitative interviews after already commencing self-administration of vaginal suppositories. Despite this, a sizeable minority of participants in the VP Trial still noted a preference for injectable progesterone without having had previous experience taking it, highlighting the possibility that allowing women a choice between comparable formulations could promote acceptability and adherence to antenatal progesterone use in HIV-positive women in future studies. Long-acting formulations such as a ring or implant have been shown to have the highest acceptability and adherence in microbicide studies and should similarly be considered for progesterone delivery for the prevention of preterm birth [27].

We acknowledge a number of limitations to this study. First, we elected not to interview participants who had suffered a stillbirth or neonatal death such that preterm birth was under-represented in our interviewed sample. While we made this decision to avoid provoking feelings of guilt or liability for these adverse outcomes, this may have biased our results surrounding acceptability but is less likely to have affected emergent themes around product adherence. Second, we did not interview women who declined participation in the VP trial so that barriers to study participation or product use likely were not representative of the general population. Additionally, because interviews were carried out in the study clinic and sometimes at the time of other routine study procedures, participants may have emphasized positive aspects of their experience over barriers and challenges. While the interviewer was not a member of the regular VP Trial staff, we cannot exclude the possibility that desirability bias may have affected our findings. Third, due to overall high adherence in the pilot study, we purposively over-sampled participants who experienced adherence challenges to increase the likelihood of capturing barriers. Because of this, while the sample size was designed to reach thematic saturation, some barriers to adherence may not have emerged in the pilot trial. Additionally, given that questionnaires and interviews were conducted either in the late third trimester or postpartum, participant recall of facilitators and barriers to product use may have been imperfect. Finally, the VOICE-C study noted that women randomized to serial ethnographic interviews were more candid in later sessions about non-adherence to the study product [7], which introduces the possibility of reticence among our participants, who each underwent a single semi-structured interview. In a larger study, longitudinal interviews through the course of study participation could be employed to overcome any early reticence.

## Conclusions

In summary, in this mixed methods study we found key influences on acceptability of a randomized trial of VP to prevent PTB among HIV-infected women in Zambia. A full-scale trial should employ methods to encourage uptake and adherence and to reduce negative perceptions towards trial participation.

## Supporting information

S1 File Questionnaire. (PDF)

S2 File Interview guide. (PDF)
