# Correction: Acceptability of a trial of vaginal progesterone for the prevention of preterm birth among HIV-infected women in Lusaka, Zambia: A mixed methods study

**DOI:** 10.1371/journal.pone.0246489

**Published:** 2021-01-28

**Authors:** The PLOS ONE Staff

## Notice of republication

This article was republished on December 11, 2020, to correct errors in the Qualitative interviews subsection of the Results section that were introduced during the typesetting process. The publisher apologizes for the errors. Please download this article again to view the correct version. The originally published, uncorrected article and the republished, corrected articles are provided here for reference.

## Supporting information

S1 File Originally published, uncorrected article. (PDF)

S2 File Republished, corrected article. (PDF)

## References

[1] Price JT, Mabula-Bwalya CM, Freeman BL, Carda-Auten J, Phiri WM, Chibwe K, et al (2020) Acceptability of a trial of vaginal progesterone for the prevention of preterm birth among HIV-infected women in Lusaka, Zambia: A mixed methods study. PLoS ONE 15(9): e0238748 10.1371/journal.pone.0238748 32970697 PMC7514015

