# Supplementary material for: Acceptability of a trial of vaginal progesterone for the prevention of preterm birth among HIV-infected women in Lusaka, Zambia: A mixed methods study
Source: PLoS One. 2020 Sep 24;15(9):e0238748. doi: 10.1371/journal.pone.0238748 (PMC7514015; doi:10.1371/journal.pone.0238748)
Supplement: S1 File — (PDF) [file pone.0238748.s001.pdf]

VP STUDY EXIT FORM

Place PTID Sticker Here

Check: [ ][ ]

Today's Date: __ __ / __ __ / __ __ __ __ DD/MM/YYYY

EGA today: __ __ weeks __ days ○ No longer pregnant

Visit Number: __ __ . __ __

1. Is the participant still using the medication? ○ No ○ Yes **[SKIP TO Q4]**

2. Date the participant stopped taking the medication: __ __ / __ __ / __ __ __ __ DD/MM/YYYY
   ○ Unknown

3. If UNKNOWN, gestational age when she stopped taking the medication: __ months ○ Unknown

4. What [is/was] the hardest part about taking the medication? (*Mark only ONE answer*)
   - ○ Difficulty remembering to take it
   - ○ Didn't like using the product
   - ○ Couldn't get to clinic to get refills because of transportation issues
   - ○ Couldn't get to clinic to get refills because of work or school
   - ○ Couldn't get to clinic to get refills because of childcare or other household duties
   - ○ Partner or family member did not know or did not want her to be in the study
   - ○ Other: ____
   - ○ Doesn't know
   - ○ No response / Nothing was hard

Please have the participant rate the following statements (*use the faces to assist)*:

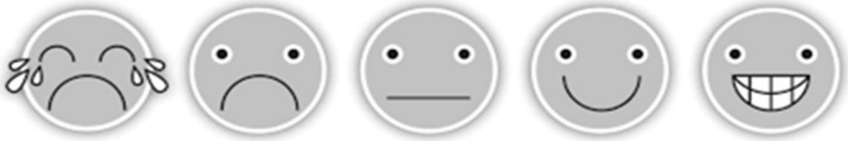

Strongly Disagree | Disagree | Neutral | Agree | Strongly Agree

5. I am happy that I took part in this study.
   ○ Strongly Disagree ○ Disagree ○ Neutral ○ Agree ○ Strongly Agree

6. I did not mind taking the vaginal medication once a day.
   ○ Strongly Disagree ○ Disagree ○ Neutral ○ Agree ○ Strongly Agree

7. If I had a choice between a vaginal medication that I give myself every day and a shot in my arm once a week to prevent an early birth, I would prefer to take the vaginal medication.
   ○ Strongly Disagree ○ Disagree ○ Neutral ○ Agree ○ Strongly Agree

8. Women in my community would like to take a medication during pregnancy to prevent them from having an early birth.
   ○ Strongly Disagree ○ Disagree ○ Neutral ○ Agree ○ Strongly Agree

Completed by: __ __ __ Date: __ __ / __ __ / __ __ __ __ DD/MM/YYYY

Checked by: __ __ __ Date: __ __ / __ __ / __ __ __ __ DD/MM/YYYY

Entered by: __ __ __ Date: __ __ / __ __ / __ __ __ __ DD/MM/YYYY

11675768
