# Supplementary material for: Acceptability of a trial of vaginal progesterone for the prevention of preterm birth among HIV-infected women in Lusaka, Zambia: A mixed methods study
Source: PLoS One. 2020 Sep 24;15(9):e0238748. doi: 10.1371/journal.pone.0238748 (PMC7514015; doi:10.1371/journal.pone.0238748)
Supplement: S2 File — (PDF) [file pone.0238748.s002.pdf]

Interview Guide for Interviews with Women Enrolled in Trial

Introduction

Hello, my name is _______ and I'm working with University of North Carolina – Global Projects Zambia (UNC-GPZ) organization. Thank you for agreeing to talk to me today. As part of this study, we are interested in hearing your experiences with taking part in this research study about preterm birth. **Preterm birth is when a woman delivers her baby too early.** We are hoping that by understanding what women go through when they take part in research, we can improve the way we do studies in the future.

I would like to audio record the interview, so I don't miss anything that you say and so that later I can be sure that I don't misunderstand what you said. The recording of this interview will be written down and then the tapes will be destroyed. Your name will not be included in the audio recording or on any of the documents so that no one will know that you took part in this interview. Your answers will be kept confidential and secure. Is it okay if I audio record our discussion today?

Thank you.

Your participation is completely voluntary. If you want to stop at any time or do not want to answer a question that I ask, just tell me. That is not a problem. I am very interested in your thoughts and opinions. There are no correct or incorrect answers. Please feel free to say whatever you think. The interview will last about 30 minutes. Do you have any questions before we start?

1. Now that you have taken the study medication for some time, can you tell me what it was like for you to use the vaginal medication?
   Probe:
   - *How did you feel about taking the medication every night?*

Follow up: Was there anything that made using the medication easy or difficult for you?
Probe:
- *Did you notice anything difficult or inconveniencing about using the medication?*
- *Did you find anything helpful about the medication?*
- *In what ways, if any, did using the medication disrupt any of your normal habits/behaviors?*
- *In what ways, if any, did using this medication disrupt any of your normal behaviors with your partner?*
- *Was it easy or difficult to understand the instructions on how to use the applicators?*

2. Please list for me what things you did, if anything, to remember to take your medications every time you were supposed to.
   Probe:
   - *What helped you remember to take your medication every time you are supposed to?*

Follow-up: You mentioned X as one thing you did to remember to take your medicines, was there anything else?

Follow-up: You mentioned X and Y, anything else?

1. ____
2. ____
3. ____
4. ____
5. ____

3. Now please list for me anything that you can think of that either you or the study staff could have done to make it easier to help you remember to take your medication as prescribed?

Follow-up: You mentioned X as one thing that could have helped you better remember to take your medicines, is there anything else?

Follow-up: You mentioned X and Y, is there anything else?

1. ____
2. ____
3. ____
4. ____
5. ____

4. How would you feel about getting an injection/shot of this medication [progesterone] in your arm once a week throughout your pregnancy to prevent the baby from coming too early?
   Probe:
   - *If you were offered an option of using the vaginal medication or getting an injection each week, which would you prefer?*
   - *What is/are the reason(s) you would choose that one over the other?*
   - *Would most women in your community prefer an injection or a vaginal tablet more?*

5. What are your experiences with scheduling and coming to the clinic for study visits?
   Probe:
   - *Can you tell me about specific things that made it either easy or difficult to come to your visits?*

Follow-up: Is there anything the study staff could have done to make it easier for you to come to these visits?

6. Please tell me about bringing your used applicators back to the clinic. How easy or difficult was it for you?
   Probe:
   - *What made it easy or difficult for you to bring your used applicators back?*

Follow-up: Is there anything the study staff could have done to make it easier for you to bring your used applicators back?

7. What kind of experiences have you heard from other women participating in the study?

8. What could the study staff do better to improve the overall experiences of women taking part in the study?

9. Did you tell any of your friends or family members that you are taking part in this study?
   Probe:
   - *Did you tell your partner about the study?*

Follow-up: How did you decide whether to tell them?

Follow-up: What did they say when you told them about being a part of this study?
   Probe:
   - *How did they feel about you being part of the study?*

10. What are some reasons that women in your community may want to take part in research studies?
    Probe:
    - *What do women like about participating in research studies?*

11. What are some reasons that women in your community may NOT want to take part in research studies?
    Probe:
    - *What do women NOT like about participating in research studies? What do women think about studies that use a placebo?*
